# Supplementary figures and images for: Generation and diversification of recombinant monoclonal antibodies
Source: eLife. 2021 Dec 31;10:e72093. doi: 10.7554/eLife.72093 (PMC8763395; doi:10.7554/eLife.72093)

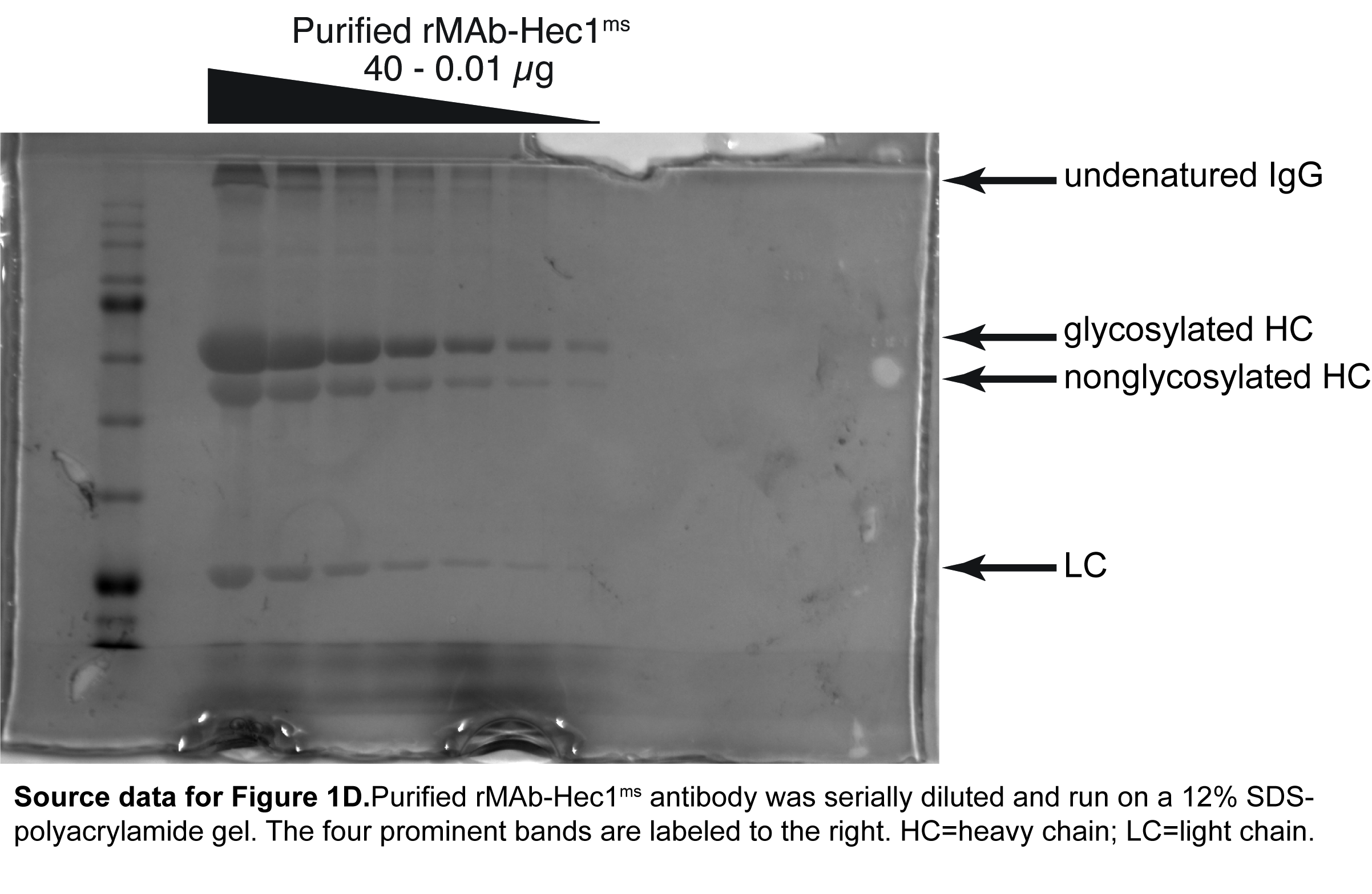

Supplement: Figure 1—source data 1. [file elife-72093-fig1-data1.tif]

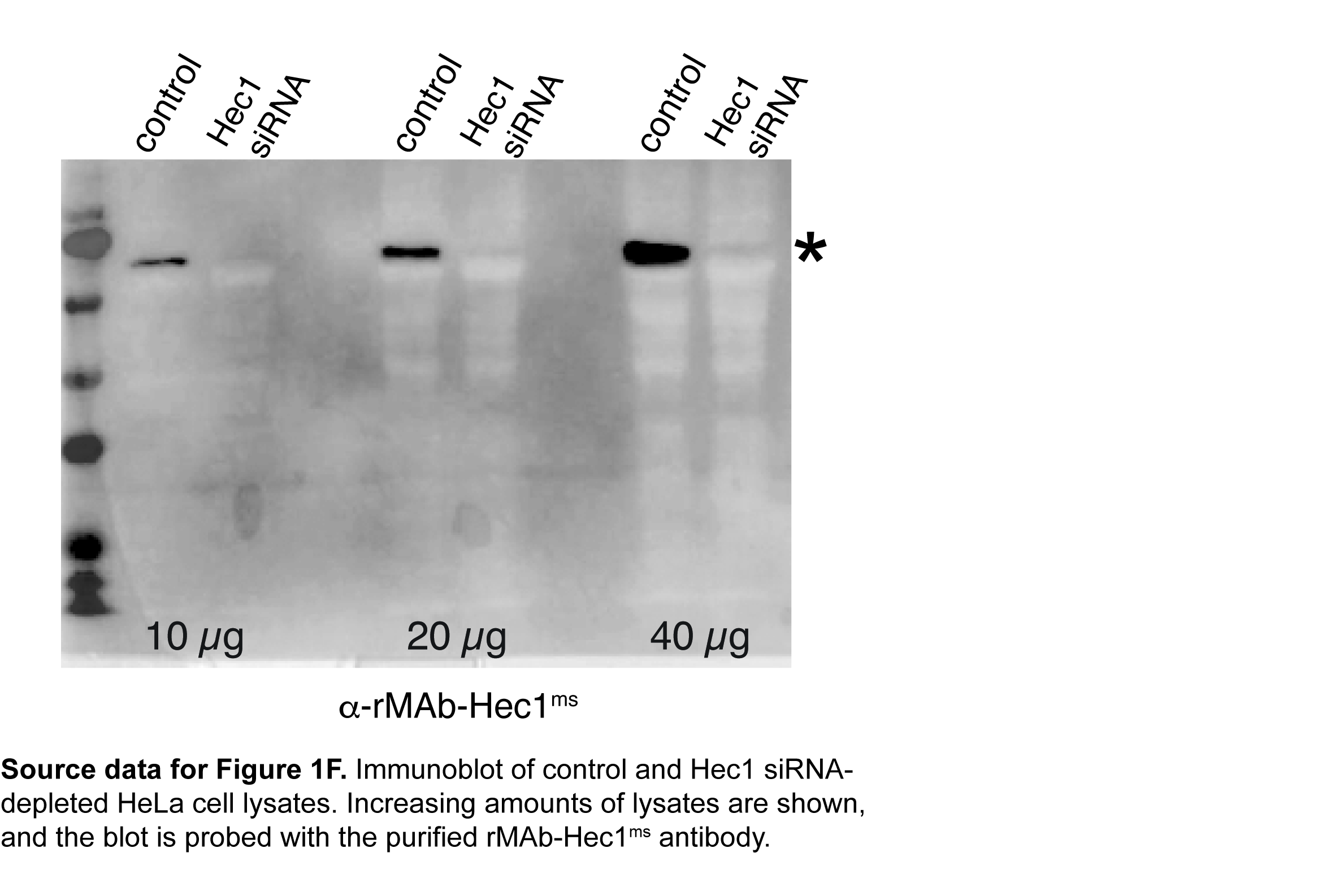

Supplement: Figure 1—source data 2. [file elife-72093-fig1-data2.tif]

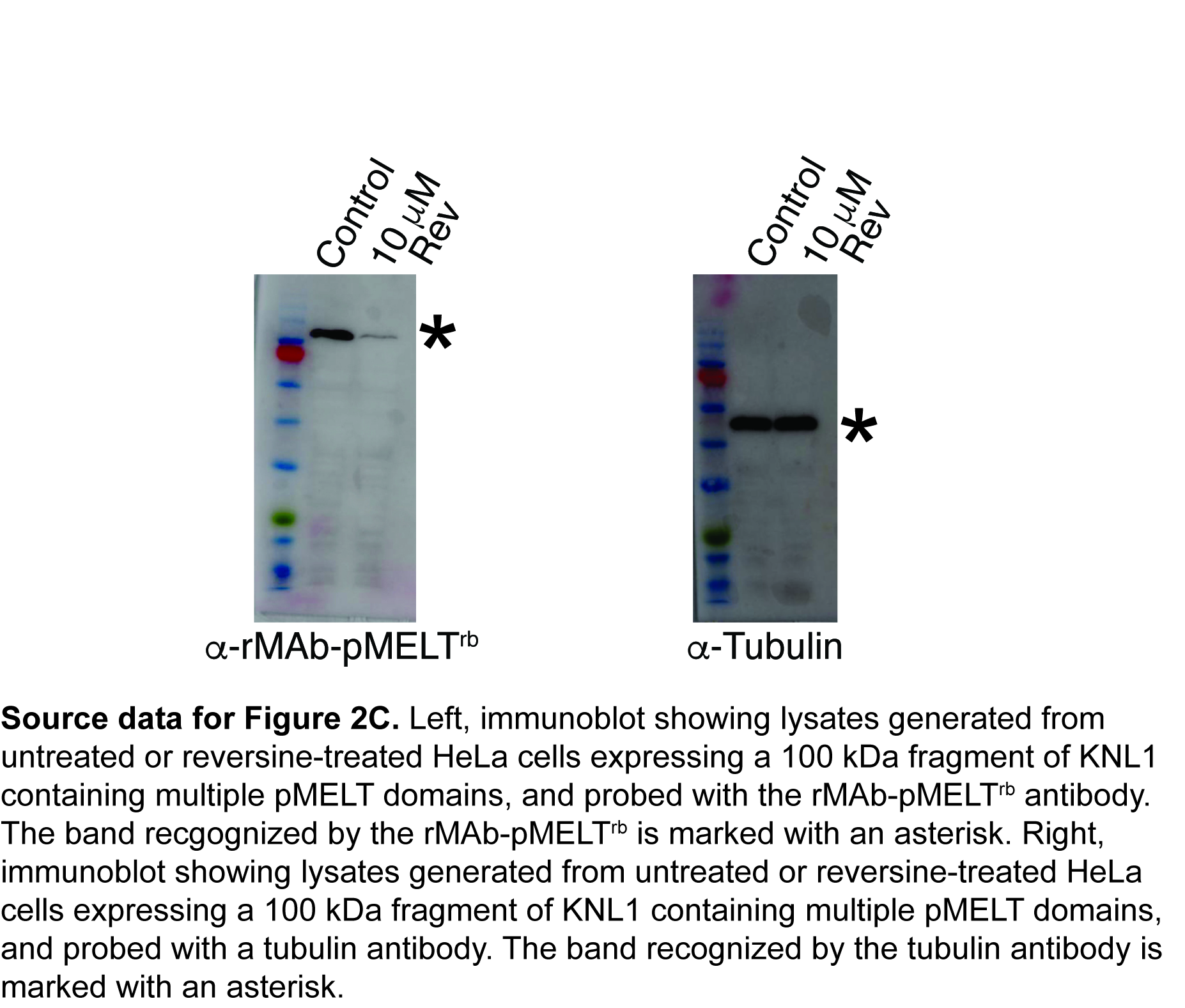

Supplement: Figure 2—source data 1. [file elife-72093-fig2-data1.tif]

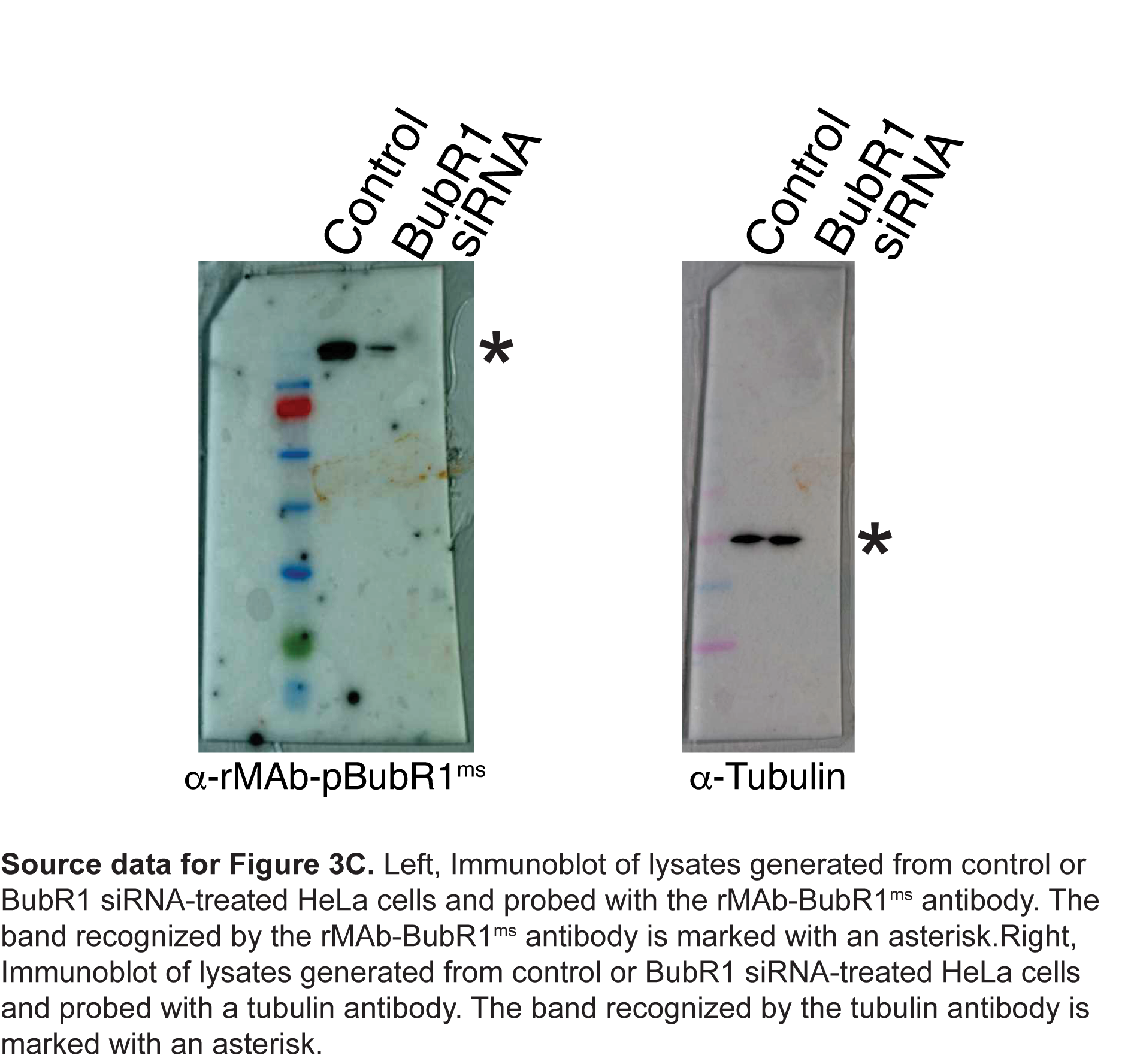

Supplement: Figure 3—source data 1. [file elife-72093-fig3-data1.tif]

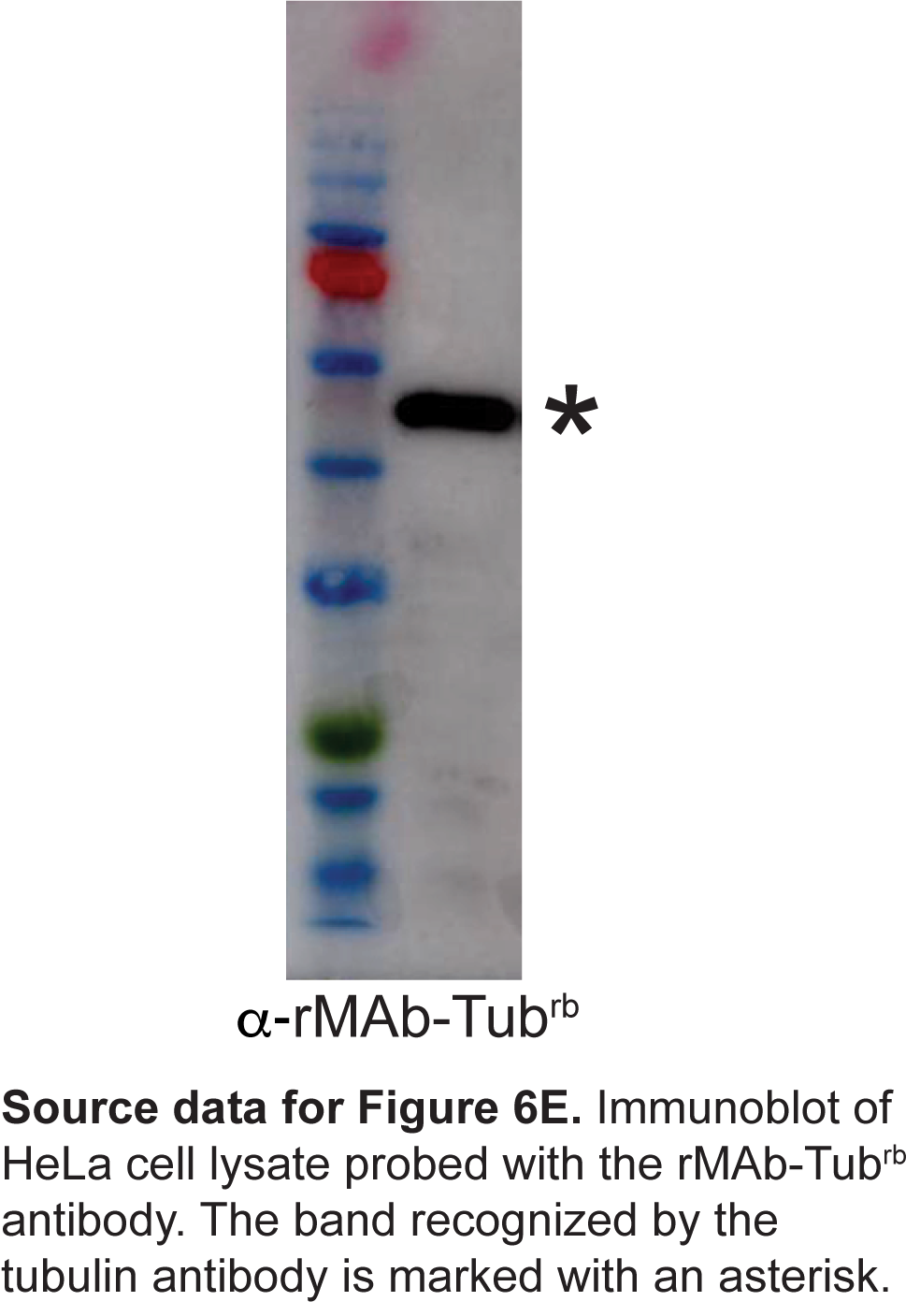

Supplement: Figure 6—source data 1. [file elife-72093-fig6-data1.tif]
